# Intravenous immunoglobulin (IVIg) provides protection against endothelial cell dysfunction and death in ischemic stroke

**DOI:** 10.1186/2040-7378-6-7

**Published:** 2014-06-20

**Authors:** Alexander Widiapradja, Tomislav Santro, Milan Basta, Christopher G Sobey, Silvia Manzanero, Thiruma V Arumugam

**Affiliations:** 1 Department of Physiology, Yong Loo Lin School of Medicine, National University of Singapore, Singapore 117597, Singapore; 2 School of Biomedical Sciences, The University of Queensland, St Lucia, QLD 4072, Australia; 3 Biovisions, Inc., Potomac, MD, USA; 4 Department of Pharmacology, Monash University, Clayton, Victoria, Australia; 5 Australian Institute for Bioengineering & Nanotechnology, The University of Queensland, St Lucia, QLD 4072, Australia

**Keywords:** Intravenous immunoglobulin, Ischemic stroke, Blood–brain barrier, Endothelium, Permeability

## Abstract

**Background:**

The brain endothelium is a key component of the blood brain barrier which is compromised following ischemia, allowing infiltration of damaging immune cells and other inflammatory molecules into the brain. Intravenous immunoglobulin (IVIg) is known to reduce infarct size in a mouse model of experimental stroke.

**Findings:**

Flow cytometry analysis showed that the protective effect of IVIg in ischemia and reperfusion injury *in vivo* is associated with reduced leukocyte infiltration, suggesting an involvement of the endothelium. In an *in vitro* model of ischemia, permeability analysis of the mouse brain endothelial cell line bEnd.3 revealed that IVIg prevented the loss of permeability caused by oxygen and glucose deprivation (OGD). In addition, western blot analysis of these brain endothelial cells showed that IVIg prevented the down-regulation of tight junction proteins claudin 5 and occludin and the decline in anti-apoptotic proteins Bcl-2 and Bcl-XL caused by OGD.

**Conclusion:**

IVIg protects endothelial cells from ischemic insult. These studies support the use of IVIg as a pharmacological intervention for stroke therapy.

## Introduction

Intravenous immunoglobulin (IVIg) is a purified concentrated human immunoglobulin solution composed primarily of IgG, obtained by fractionating blood plasma from a pool of healthy donors. Due to its immunomodulatory and anti-inflammatory effects, IVIg is used as a therapeutic modality for many immune disorders, including those that affect the nervous system [1]. We have shown that IVIg treatment reduces brain infarct volume and mortality in a mouse model of stroke [2,3]. We have also observed in *in vivo* and *in vitro* mouse models of stroke that IVIg promotes neuronal survival by inhibiting the activation of inflammasomes and apoptotic signaling and by increasing the levels of the anti-apoptotic protein B-cell lymphoma 2 (Bcl-2) [3,4].

In response to the local and systemic inflammation that occurs following stroke, the cerebral vasculature is subjected to endothelial cell activation, leukocyte-endothelial cell adhesion, blood brain barrier (BBB) dysfunction, activation of glial cells and an enhanced generation of inflammatory mediators [5]. Clinical intervention to attenuate and/or delay BBB dysfunction may improve neurological outcome and facilitate patient recovery. Animal studies have shown that administration of IVIg impairs leukocyte adhesion to endothelial cells, attenuates complement-mediated damage, modulates cytokine production by various cell types and inhibits apoptosis [2,6-8]. However, it is not known whether IVIg protects from cerebrovascular dysfunction following ischemic stroke. Endothelial permeability, the ability of the endothelial cell monolayer to restrict the transfer of solutes from the blood to the brain parenchyma, is essential for cerebrovascular function. Endothelial permeability comprises paracellular and transcellular mechanisms, and tight junctions are a key component of the paracellular pathway, which is vulnerable to ischemic injury. Therefore, measuring tight junction proteins has been shown as a valid indicator of the paracellular pathway of endothelial permeability and its damage in ischemic injury [9]. The findings of this study reveal a beneficial effect by IVIg on ischemia-induced leukocyte recruitment as well as on endothelial permeability (mediated by tight-junction proteins claudin 5 and occludin), as well as an anti-apoptotic effect on endothelial cells mediated by Bcl-2 and Bcl-XL.

## Materials and methods

### Focal cerebral ischemia/reperfusion (I/R) stroke model

Three-month-old C57BL/6 J male mice were sourced from the Animal Resources Centre, Western Australia, and subjected to transient middle cerebral artery occlusion for 1 h followed by reperfusion, as described previously [2]. Neurological deficit, the functional abnormalities observed in mice as a result of brain damage, were scored on a five-point scale: 0, no deficit; 1, failure to extend right paw; 2, circling to the right; 3, falling to the right; and 4, unable to walk spontaneously. Only animals that showed a neurological deficit score of 1 to 3, consistent with moderate stroke damage [2,5], were used. Of 24 mice, 4 mice were excluded due to lack of neurological deficit (inclusion 83.4%). 8 mice were administered 2 g/kg of IVIg (250 μl; Kiovig, Baxter) by infusion into the femoral vein 3 h after reperfusion by an independent investigator and the remaining 12 mice were administered the vehicle phosphate-buffered saline (PBS). For each experiment, each group of mice was placed in a separate box after IVIg or vehicle injection and boxes were coded to ensure blind allocation. These experiments were approved by the Animal Care and Use Committee of The University of Queensland (Australia).

### Flow cytometry

Flow cytometric analysis of immune cells was performed as previously described [5]. Animals were euthanized and perfused with PBS. Ipsilateral hemispheres were dissected, digested for 30 min at 37°C (1 mg/mL collagenase, 0.1 mg/mL DNAse I in Dulbecco's Modified Eagle's Medium, DMEM), and passed through a cell strainer. Cells were incubated with standard erythrocyte lysis buffer on ice and separated from myelin and debris by Percoll gradient (GE Healthcare) centrifugation. After staining of surface markers for CD45 (30-F11, 1:100) and CD11b (M170, 1:300), cells were fixed using fixation buffer (all eBioscience). Data were acquired with a LSR II FACS system and analyzed with FACSDiva (both BD Biosciences). Doublets were excluded with FSC-A and FSC-H linearity. 4 independent experiments were carried out on different days, each with 2 to 3 pooled animals per sample, to obtain adequate statistical power [5].

### Oxygen and glucose deprivation (OGD)

The murine brain endothelial cell line bEnd.3 (ATCC CRL-2299) was grown to confluence in DMEM supplemented with 10% fetal bovine serum, 1% penicillin/streptomycin and 1% L-glutamine (all Life Technologies). OGD was induced by exposing the cells to Locke’s buffer [3] for 10 min before incubation in an oxygen-free chamber with 95% N_2_, 5% CO_2_ for 0.5-3 h. IVIg was added at the reported concentrations and proline (Sigma Aldrich), an aminoacid with no known affinity for any cell receptors, was used at a concentration of 5 mg/mL as vehicle control for all experiments.

### Permeability assay

In order to assess the effect of IVIg on cell permeability during OGD, endothelial bEnd.3 cells were grown on 0.4 μm pore membrane cell culture inserts (Nunc) on 24 well plates. Cells were cultured on the upper side of the insert and allowed to grow to confluence. 5 mg/mL FITC-dextran (Sigma Aldrich) was added to the upper chamber containing IVIg and Locke’s buffer before exposure to OGD. After 3 h, 100 μl of media from the lower chamber was collected and the amount of FITC-dextran was measured using a fluorescence plate reader Varioskan (Thermo Scientific) at absorbance and emission wavelengths of 492 nm and 520 nm respectively.

### Lactate dehydrogenase assay

The bEnd.3 cells were cultured in 24 well plates until confluent before OGD treatment in the presence or absence of IVIg for 0.5, 1, 2 or 3 h. The LDH assay was performed according to the manufacturer’s instructions (Roche), except that samples were incubated with the reaction solution for 1 h. The reaction was stopped by adding 1 N HCl, and absorbance was measured on the different sample wells. Each sample measurement was carried out in triplicate.

### Immunoblotting

Protein samples were run for sodium dodecyl sulfate–polyacrylamide (10%) gel electrophoresis and then electro-blotted onto a nitrocellulose membrane. The membrane was blocked for 1 h and incubated overnight at 4°C with antibodies selective for occludin (Life Technologies), claudin 5 (Life Technologies), JAM-A (Santa Cruz), ZO-1 (Abcam), AIF (Cell Signaling), Bcl-2 (Cell Signaling), Bcl-XL (Cell Signaling) and β-actin (Sigma Aldrich). After washing, the membrane was incubated with secondary antibodies for 1 h, washed again and incubated with substrate for enhanced chemiluminescence (Pierce). After visualization on X-ray films (Fujifilm), protein levels were quantified by densitometry using Image J software.

### Immunocytochemistry

bEnd.3 cells were cultured on microscope coverslips, exposed to OGD and treated with IVIg or vehicle, and fixed in 4% paraformaldehyde. Incubation for 1 h with claudin 5 (Cell Signaling) antibody was followed by incubation with Alexa fluor 488 conjugated secondary antibody (Life Technologies). Nuclei were stained with DAPI and mounted with VectaShield (Vector). Images were acquired using a fluorescence microscope (Olympus).

### Data analysis

Statistical power was calculated using an expected 50% reduction in inflammatory infiltrate and permeability, and a 50% increase in protein levels between vehicle and IVIg treated samples, based on previous results from our laboratory [2-4]. All the results are reported as means ± SEM. Overall data significance was examined by one-way analysis of variance (ANOVA). The differences between groups were considered significant at p < 0.05 using Bonferroni correction for multiple comparisons.

## Results

### IVIg treatment reduces leukocyte infiltration and protects against OGD-induced brain endothelial damage

**Figure 1:**
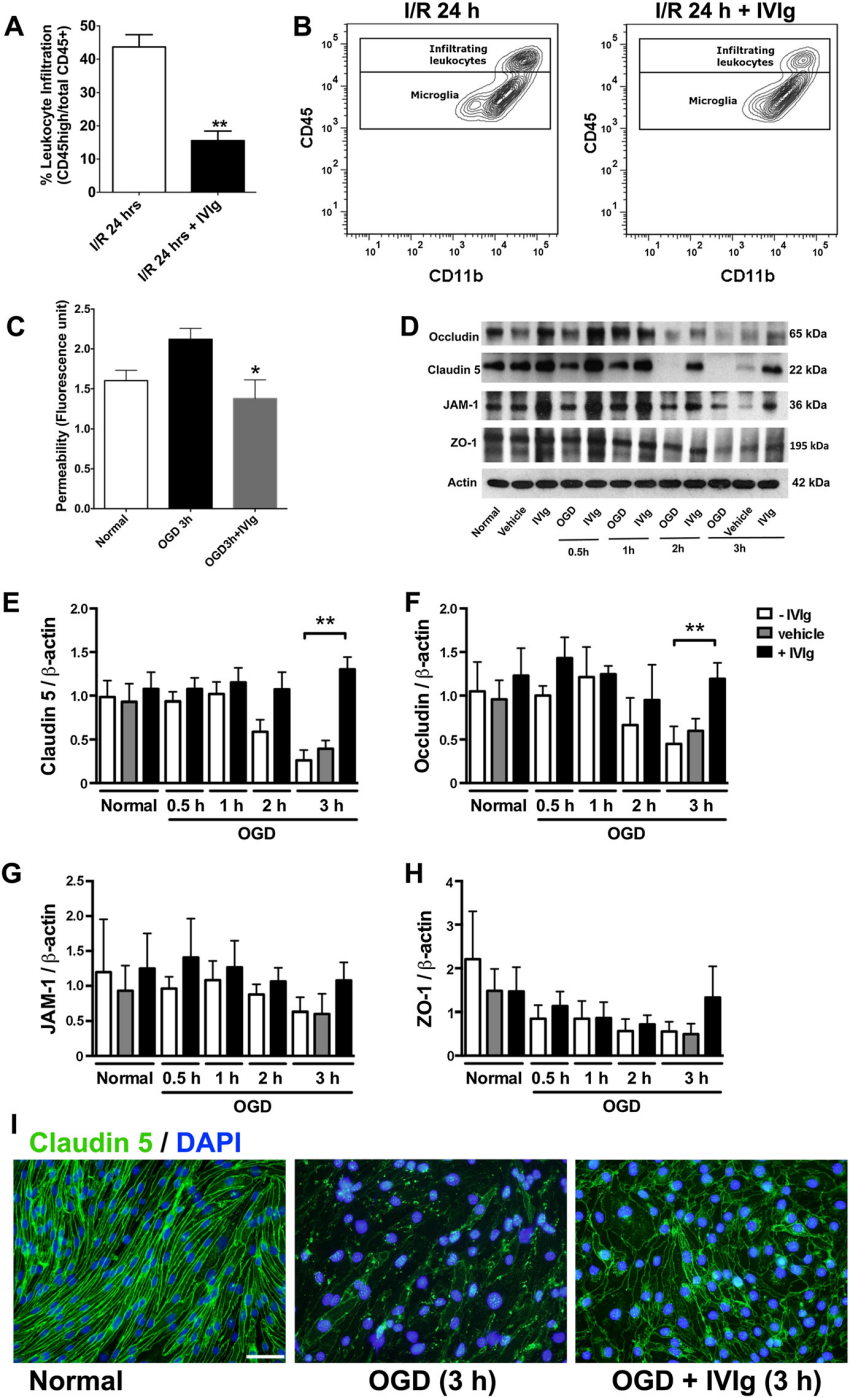
**IVIg prevents post-ischemic leukocyte infiltration and maintains blood brain barrier integrity. (A)** Administration of IVIg (2 g/kg) decreased leukocyte infiltration following 1 h and 23 h respectively of cerebral ischemia and reperfusion (I/R). ^**^p < 0.01 compared to I/R mice, n = 8-12. **(B)** Representative flow cytometry plots showing the two studied populations of CD45^+^ cells, infiltrating leukocytes (CD45 high) and microglia (CD45 intermediate). **(C)** Treatment with high concentration of IVIg (5 mg/mL) reduced permeability of bEnd.3 cell monolayers subjected to oxygen and glucose deprivation (OGD). ^*^p < 0.05 compared to OGD 3 h. **(D, E and F)** IVIg rescues the decrease in claudin 5 and occludin levels that occurs after 3 h OGD in untreated or vehicle controls. ^**^p < 0.01. **(G and H)** A similar trend was seen in the expression levels of JAM-1 and ZO-1, however statistical significance was not reached. **(I)** Immunofluorescence staining showed the restoration of claudin 5 expression in IVIg-treated bEnd.3 cells subjected to 3 h OGD. Scale bar: 20 μm.

Ischemic stroke promotes leukocyte recruitment to the injured tissue [5]. The first aim was to determine whether the IVIg dose previously shown as optimal to reduce infarct size 72 h after ischemia and reperfusion in this mouse model [2] was able to decrease leukocyte infiltration to the ischemic hemisphere at 24 h. The chosen time point of IVIg infusion was 3 h post-reperfusion, as this was the latest time point previously shown to reduce infarct size [2], and late intervention supports clinical application. Our data showed that presence of CD45^high^ immune cells in the ipsilateral hemisphere 24 h post-ischemia was attenuated in IVIg-treated mice compared to vehicle-treated controls (Figure 1A and B). Since tight regulation of endothelial permeability is pivotal to BBB integrity, we next assessed the effect of IVIg on permeability using the mouse brain endothelial cell line bEnd.3, measured as the ability for FITC-dextran to cross the cell monolayer 3 h after oxygen and glucose deprivation (OGD). IVIg-treated cells exhibited reduced permeability compared with control cells (Figure 1C). In order to investigate whether this was a consequence of tight junction protein modulation, we assessed expression levels of claudin 5, occludin, junctional adhesion molecule 1 (JAM-1) and zona occludens-1 (ZO-1). Immunoblot analyses showed that expression of each of these tight junction proteins was reduced after 3 h OGD (Figure 1D-H). However, the decline in claudin 5 and occludin levels was completely rescued in the presence of IVIg (Figure 1E and F). Immunocytochemistry confirmed the disappearance of claudin 5 from the membranes of OGD-treated endothelial cells and the conservation of claudin 5 integrity in the presence of IVIg (Figure 1I). Overall these results point to a beneficial effect of IVIg on the maintenance of tight-junction proteins claudin 5 and occludin during ischemic stress, which would strengthen the BBB and contribute to a decreased leukocyte infiltration after ischemia.

### IVIg protects brain endothelial cells against OGD-induced cell death by up-regulating anti-apoptotic proteins

**Figure 2:**
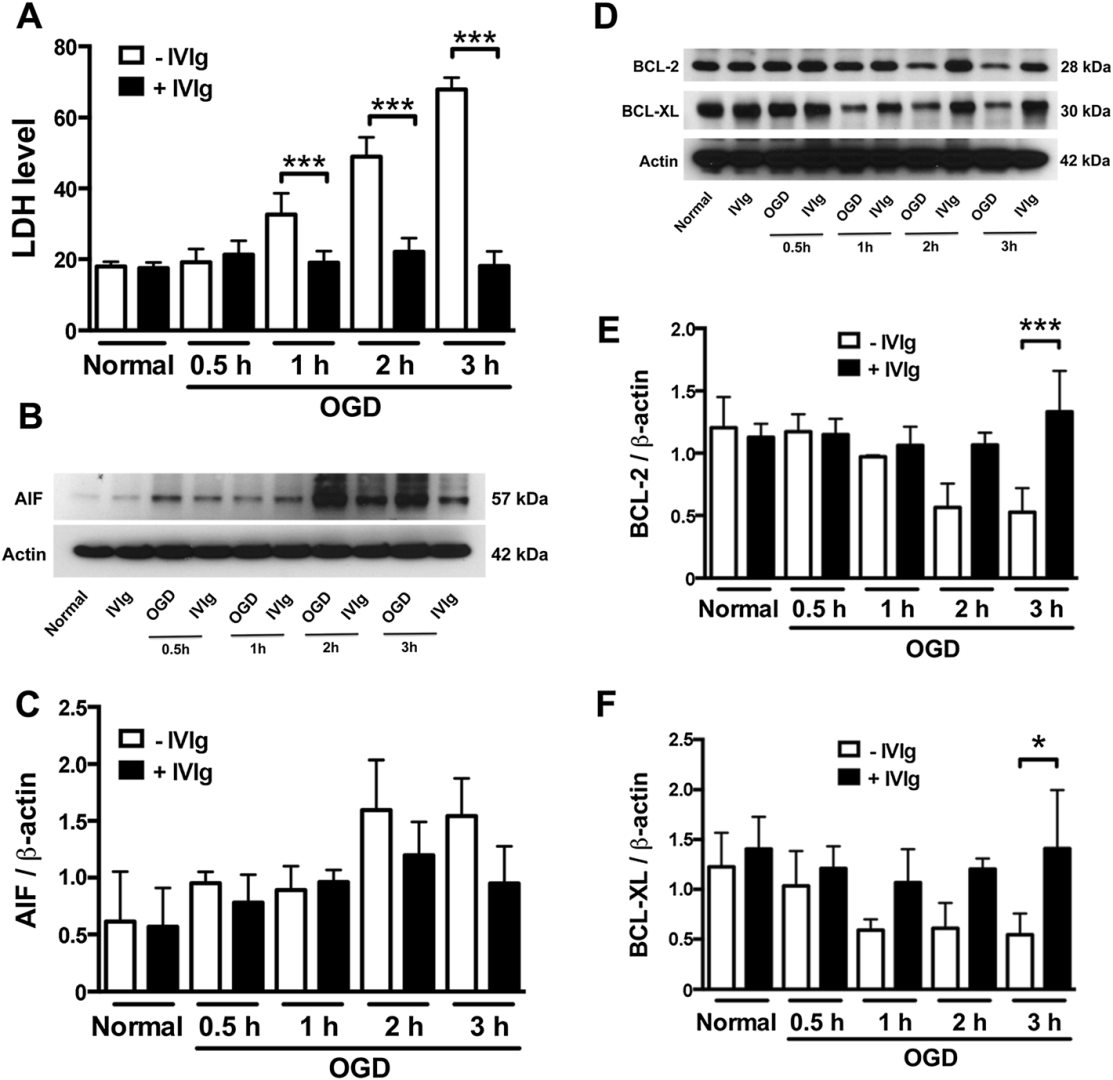
**IVIg promotes endothelial cell survival in OGD*. *(A)** IVIg treatment reduced LDH release from bEnd.3 cells subjected to 1–3 h OGD. ^***^ p < 0.001 as indicated by bars. **(B and C)** IVIg moderated the increase in AIF levels in bEnd.3 cells, however statistical significance was not reached. **(D, E and F)** IVIg prevented the decrease in Bcl-2 and Bcl-XL levels measured in bEnd.3 lysates following 3 h OGD. ^***^ p < 0.001, ^*^ p < 0.05 as indicated by bars.

Next, we investigated the effect of IVIg on the survival of brain endothelial cells during OGD. bEnd.3 cells treated with IVIg showed consistently low levels of LDH release throughout an OGD time course up to 3 h, indicative of a protective effect of IVIg against OGD-induced cell death (Figure 2A). In order to better understand the underlying mechanisms, analyses of apoptosis inducing factor (AIF), Bcl-2, and Bcl-XL were carried out by immunoblotting. IVIg treatment tended to reduce the increase in AIF observed following 2–3 h OGD, however this effect did not reach statistical significance (Figure 2B and 2C). However, IVIg significantly prevented the OGD-induced decreases of both Bcl-2 and Bcl-XL observed in untreated cultures (Figure 2D-F). These data support an action of IVIg to protect cerebral endothelial cells from undergoing ischemia-induced apoptosis.

## Discussion

We [2,3] and others [10,11] have previously demonstrated that IVIg treatment reduces brain infarct volume and mortality in experimental rodent models of stroke. This study reveals a protective effect of IVIg against endothelial dysfunction following ischemic stroke, in association with reduced leukocyte infiltration *in vivo*. The data indicate that IVIg provides a two-way protective mechanism for cerebral endothelial cells, firstly by blocking the increase in BBB permeability involving downregulation of tight-junction proteins claudin 5 and occludin, and secondly by preventing ischemia-induced endothelial cell death involving a reduction in anti-apoptotic proteins, Bcl-2 and Bcl-XL.

This study shows for the first time that infusion of a high dose of IVIg (2 g/kg body weight) 3 h after reperfusion reduces leukocyte infiltration into the brain. Ischemic stroke causes endothelial cell dysfunction with the breakdown of tight junction proteins and compromised BBB function, leading to increased protein extravasation, interstitial edema and the adhesion and transmigration of leukocytes [12]. We have previously demonstrated the injurious role of infiltrating immune cells in the pathogenesis of post-ischemic brain injury in mice [5], and the beneficial effects of IVIg treatment on the levels of inflammatory endothelial and leukocyte adhesion molecules such as ICAM-1, CD11a, and CD11b in a mouse model of ischemic stroke [2].

Ischemic stroke compromises BBB integrity, which is mediated by cytoskeleton rearrangement, and redistribution and disappearance of tight junction proteins such as claudin 5 and occludin in brain endothelial cells, resulting in increased BBB permeability [13]. Our results show that endothelial permeability, which is vulnerable to OGD, remains unaffected by OGD in the presence of IVIg. In accordance with this finding, the loss of key tight-junction proteins claudin 5 and occludin in OGD does not occur when IVIg is present. IVIg could, therefore, prevent the loss of tight junction integrity in the brain and consequently protect the BBB whose breakdown contributes to a large part of stroke-induced injury. A recent study investigating the effect of IVIg on BBB integrity in a sepsis model suggested that immunoglobulin G, A, or M improved the integrity of the BBB and inhibited symptoms of sickness in rats [14]. We previously reported that IVIg crosses the BBB and exerts protective effects in a mouse model of stroke [2,3]. There are two ways in which IVIg may be able to reduce BBB permeability and at the same time be able to cross the BBB – while IVIg may cross the intact BBB, as reported recently in mouse [15], it is also possible that IVIg crosses the damaged BBB and then exerts its permeability modulation once in the brain parenchyma.

This study highlights a previously unrecognised beneficial action of IVIg on cerebral endothelial cells exposed to ischemia, in that cell death is inhibited and levels of Bcl-2 and Bcl-XL are preserved. Bcl-2 and Bcl-XL play pivotal roles in determining cell survival or death under conditions of stress, and have both been shown to prevent endothelial cell death [16]. Ischemic conditions, or exposure to Aβ, promote a decline in Bcl-2 levels and subsequently increase apoptosis in mouse primary cortical neurons, but the presence of IVIg maintains high Bcl-2 levels and low cell death rates [3]. In physiological terms, IVIg could potentially be responsible for the survival of a number of vessels in the penumbra that would otherwise undergo cell death from ischemia, hence contributing to the extension of the infarct. Taken together, these data indicate that IVIg has significant protective characteristics for inhibiting endothelial, as well as neuronal cell death following ischemic stroke. In addition to the known neuroprotective role of IVIg, we have demonstrated that IVIg acts through multiple mechanisms to protect brain tissue after stroke. Specifically, our present findings provide evidence that IVIg inhibits endothelial dysfunction and death and reduces leukocyte infiltration following ischemic stroke.

The current view in stroke treatment supports the notion of stroke therapies that affect multiple injury mechanisms simultaneously. IVIg, with its effects on neurons, inflammatory cells and endothelium fits this definition perfectly and as such promises to be a relevant candidate for translational research to the clinic.

## Competing interests

The authors declare that they have no competing interests.

## Authors’ contributions

AW, CGS, MB and TVA conceived the study; TVA and SM coordinated the study; AW carried out protein expression studies and cell death assays with TS help; AW and TS carried out permeability assays; SM, AW, TS and TVA carried out flow cytometry; AW, SM, TVA, CS and MB wrote the manuscript. All authors read and approved the final manuscript.
